# Exploiting Antitumor Immunotherapeutic Novel Strategies by Deciphering the Cross Talk between Invariant NKT Cells and Dendritic Cells

**DOI:** 10.3389/fimmu.2017.00886

**Published:** 2017-07-31

**Authors:** Shin-ichiro Fujii, Kanako Shimizu

**Affiliations:** ^1^Laboratory for Immunotherapy, RIKEN Center for Integrative Medical Sciences (IMS), Yokohama, Japan

**Keywords:** dendritic cells, invariant NKT cells, innate immunity, adaptive immunity, cross presentation, immunotherapy

## Abstract

Immune checkpoint blockade therapy has prevailed for several types of cancer; however, its effectiveness as a single therapy is still limited. In principle, dendritic cells (DCs) should be able to control the post-therapy immune response, in particular since they can link the two major arms of the immune system: innate and adaptive immunity. Therefore, DCs would be a logical and ideal target for the development of immunotherapies. Since DCs are not activated in the steady state, an adjuvant to convert their function from tolerogenic to immunogenic would be desirable. Upon ligand activation, invariant natural killer T (iNKT) cells simultaneously activate NK cells and also energize the DCs, resulting in their full maturation. To utilize such iNKT-licensed “fully” matured DCs as adjuvants, mechanisms of both intercellular communication between DC subsets and iNKT cells and intracellular molecular signaling in DCs have to be clarified and optimized. To generate both innate and adaptive immunity against cancer, a variety of strategies with the potential to target iNKT-licensed DCs *in situ* have been studied. The benchmark of success in these studies, each with distinct approaches, will be the development of functional NK cells and cytotoxic T cells (CTLs) as well as generation of long-term, memory CTL. In this review, we provide a framework for NKT-mediated immunotherapy through selective DC targeting *in situ*, describe progress in the design of licensed therapies for iNKT cell targeting of DCs, and highlight the challenge to provide maximal benefit to patients.

## Introduction

Invariant natural killer T (iNKT) cells have several distinguishing characteristics, most notably they express an invariant TCRα chain, Vα14Jα18 in mice and Vα24Jα18 in human, paired with a TCRβ chain of limited diversity (1, 2). Unlike most αβTCRs, which recognize peptide MHC I/II complexes, iNKT TCRs recognize glycolipid antigens presented by the MHC class I-like molecule CD1d (3–5). The first NKT glycolipid ligand was identified by Kirin Pharmaceuticals (6). They extracted agelasphins as glycosphingolipid compounds from a marine sponge called *Agelas mauritianus*. They modified the structure of this compound and established a synthetic ligand with a branched galactosylceramide, commonly referred to as α-GalCer (7, 8). In addition to α-GalCer, iNKT cells recognize certain microbial ligands, for example cell wall sphingolipids from *Sphingomonas, Borrelia*, or *Streptococcus* (9–11). iNKT cells recognize such natural or synthetic glycolipids and promptly produce a broad range of cytokines. iNKT cells are not only stimulated by these glycolipid ligands directly *via* their invariant TCR but also indirectly. Since iNKT cells express IL-12 receptors, they can be stimulated by IL-12 released from dendritic cells (DCs) or macrophages. For example, *Salmonella typhimurium* does not express a glycolipid ligand, but can stimulate iNKT cells *in vivo*. In this case, the iNKT cells can be activated by both IL-12 and recognition of endogenous glycosphingolipid ligand on DCs (1, 12).

In the course of establishment or recurrence of tumor cells, genetic factors or immune related pressure may mediate the selective outgrowth of tumor cell clones lacking MHC or potentially immunogenic tumor associated antigens (TAA), thus leading to heterogeneous tumor cell evolution (13). In terms of cancer immunity, tumors are in general composed of two types of cells, some are MHC positive, but others are MHC negative. Based on our current understanding, the former can be eliminated by cytotoxic T cells (CTLs) and the latter can be eliminated by NK cells, thus both innate and adaptive immune responses are required for complete elimination of tumors. It is well-known that DCs can play a crucial role in activating both innate and adaptive immune responses (14–16). We and others demonstrated that iNKT cells, as well as many toll-like receptor (TLR) ligands, can be used for activation of DCs to bridge innate and adaptive immunity (17–20). In this review, we detail the rationale for modulation or optimization of iNKT cell-licensed antigen-expressing DCs and also describe various attempts that have so far been made for developing antitumor therapeutic strategies.

## NKT Cell Subsets—Localization and Function

When TCRs on iNKT cells are stimulated by a ligand such as α-GalCer, they are capable of producing IFN-γ and IL-4. These Vα14^+^iNKT cells were recently shown to belong to three distinct functional subsets. The NKT1, NKT2, and NKT17 cells all express promyelocytic leukemia zinc finger, but can be distinguished because they are mainly regulated by transcription factors similar to those of helper T cells, i.e., T-bet, GATA-3, and RORγt, respectively (21, 22). Development of each type of iNKT cell is generally related to the cytokine milieu encountered upon activation (IFN-γ, IL-4, or IL-17). An intravenous injection of α-GalCer rapidly activates NKT1 cells in the red pulp of the spleen and liver, thus leading to systemic IFN-γ and IL-4 responses, but not iNKT cells in LN and thymus (23, 24). NKT2 cells are mainly located in the medullary area of the thymus and T cell zone of the spleen, as well as in mesenteric LNs. Oral administration of α-GalCer may induce the activation of NKT2 cells in mLN, resulting in local IL-4 production (23, 24). NKT17 cells which are capable of producing IL-17, but not IFN-γ, are particularly enriched in the lung and the subcapsular region of LNs (23, 24). Thus, the pattern and amount of cytokine production can be determined by the location of NKT cells, NKT cell type and routes of administration of NKT ligands.

## Induction of NK Cell Responses as an Adjunctive Effect of iNKT Cell Therapy

The critical role of iNKT cells in tumor immunosurveillance was shown in chemically induced spontaneous tumor models (25). The transfer of iNKT cells prevented the induction of methylcholanthrene sarcoma tumors in Jα18^−/−^, iNKT cell-deficient mice. A similar protocol involving transfer of iNKT cells had demonstrated a significant antitumor effect in p53-deficient mice (26) and in the transgenic adenocarcinoma of the mouse prostate tumor model (27).

**Figure 1:**
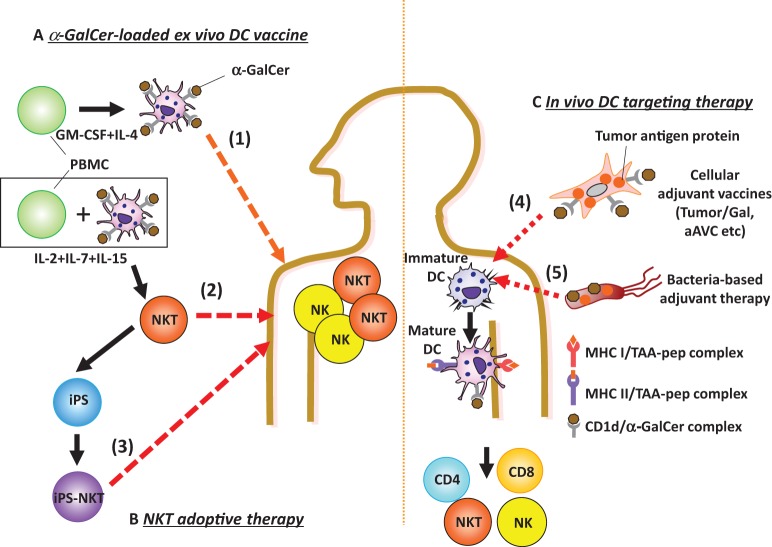
*Ex vivo* or *in vivo* glycolipid-based dendritic cell (DC) immunotherapy. **(A,B)** *Ex vivo* glycolipid-based DC therapy and NKT transfer therapy have been studied. **(A)** (1) Active immunization with *ex vivo* DCs: monocyte-derived DCs loaded with α-GalCer (DCs/Gal) or autologous PBMCs pulsed with α-GalCer are administered intravenously to cancer patients. The invariant natural killer T (iNKT) and NK cells are promptly activated in lung, liver, and spleen. **(B)** As passive immunization, effector cells are adoptively transferred. (2) For this approach, *ex vivo* iNKT cells are harvested after coculturing with autologous DC/Gal and then injected into cancer patients. (3) In the future, iPS-reprogrammed iNKT cells may be applicable for adoptive transfer therapy. **(C)** As new strategies of *in vivo* DC targeting therapies, (4) adjuvant vector cells, including tumor cells loaded with α-GalCer (Tumor/Gal) or tumor antigen mRNA-transfected, allogeneic CD1d^+^ cells loaded with α-GalCer (aAVC) or (5) non-somatic cell adjuvant (bacteria) will be candidates for the iNKT-triggered immunotherapy. When these agents are injected, both iNKT and NK cells will be activated. Host DCs can then prime antigen-specific CD4^+^ and/or CD8^+^ T cells.

**Figure 2:**
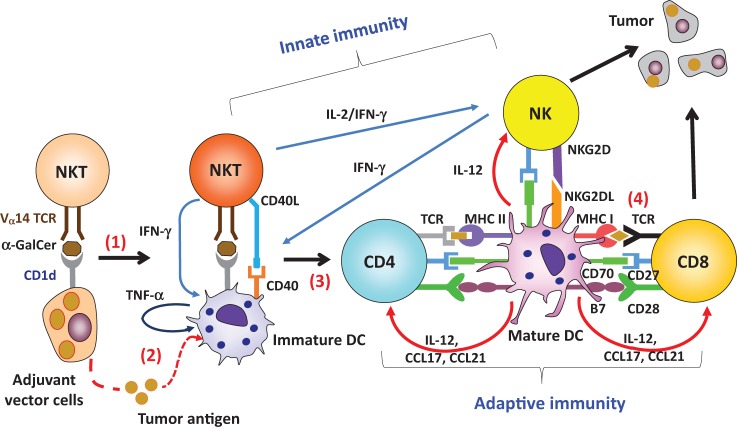
Adjuvant effect by invariant natural killer T (iNKT) cell-triggered dendritic cells (DCs) on protective antitumor responses. (1) Administration of adjuvant vector cells, including Tumor/Gal or aAVC initially stimulate iNKT cells. (2) The adjuvant vector cells are killed by iNKT cells and NK cells, and then tumor antigen released from them can be captured by endogenous CD11c^+^DCs. (3) The CD11c^+^ DCs then undergo iNKT cell-induced maturation. (4) The activated DCs can then induce an antigen-specific T cell response in the lymphoid tissues. Thus, the CD11c^+^DCs *in situ* are able to cross present tumor antigen, derived from phagocytosed adjuvant vector cells, to CD4^+^ or CD8^+^ T cells in an MHC-dependent manner.

Our first approach to understanding iNKT cell immunotherapy was to compare cell therapy (e.g., administration of CD1d^+^ cells loaded with α-GalCer) versus unbound glycolipid drug therapy (e.g., administration of soluble α-GalCer) (28). An injection of bone marrow-derived *ex vivo* DCs loaded with α-GalCer (BM-DC/Gal) induced iNKT cells capable of producing IFN-γ (28) (Figure 1), and this correlated with antitumor effects in B16 melanoma lung metastasis. In contrast, the iNKT cell response to unbound α-GalCer was more rapid, but transient and then the cells became anergic (28, 29). Thus, the glycolipid has different functional effects on iNKT cells when it is injected as a free glycolipid or in association with CD1d^+^ cells. When activated by the iNKT cell ligand, IFN-γ and IL-2 production by iNKT cells enhances the activation of NK cells as iNKT–NK axis (30) (Figure 2). The interaction between iNKT cells and DCs can also enhance NK cell activity. After activation by NKT cells, DCs express NKG2D ligands and CD70, thus leading to the activation of NK cells (31). In addition, since NK cells also express IL-12R, IL-12 released from DCs enhances NK cell-mediated IFN-γ production (Figure 2). Thus, iNKT cells efficiently stimulate NK cells. The near synchronous activation of these iNKT and NK cell can account for innate resistance to susceptible tumors.

## Efficient Induction of Antitumor CTLs by iNKT Cell-Licensed DCs

*In situ* DCs activated by iNKT cells act as a cellular adjuvant for T-cell priming. The licensing of DCs by iNKT cells occurs by several molecular mechanisms. When activated iNKT cells encounter DCs *in situ*, co-stimulatory molecules on DCs are upregulated, indicative of DC maturation (32, 33). Activated iNKT cells can promote conversion of DCs from a tolerogenic to an immunogenic state. The DC surface remodeling of these mature DCs is driven by two inflammatory cytokines, TNF-α and IFN-γ. Cytokine production by the innate lymphocytes is tightly regulated by the interactions among unique cell types. TNF-α is produced by endogenous DCs, whereas IFN-γ is produced by iNKT or NK cells (Figure 2). It was previously reported that IFN-γ-producing DCs are important for priming of the gut intraepithelial lymphocyte response against intracellular parasitic infection (34). We also tested the possibility of IFN-γ production by CD11c^+^DCs after administration of α-GalCer and found that isolated CD11c^+^cells produce IFN-γ, but that these were CD11c^+^NK cells and not any DC subset (35).

Co-stimulatory molecules, CD40, CD80, and CD86 on DCs, which are important for priming T cells, are upregulated during the early phase (from 4 h). In addition, we and others recently demonstrated upregulation of CD70, 4-1-BBL, and IL-15Ra on DCs, which are important for generation of memory T cells, at the late phase (from 40 h) (36, 37).

Other innate lymphocytes, such as γδT cells (38, 39) and NK cells (40, 41) may also have adjuvant effects. They produce IFN-γ and TNF-α, thus promoting the maturation of DCs and help for the generation of CTLs. We also compared the magnitude of T cell responses after priming with the iNKT cell ligand α-GalCer versus NK cell ligands, such as retinoic acid early inducible-1ε (Rae1ε), Rae1γ, CD70 and murine UL16-binding protein-like transcript 1 (Mult-1). CTL induction triggered by iNKT cells is apparently more powerful than that triggering by NK cells (42).

An important difference between these other innate lymphocytes and iNKT cells lies in the CD40L signal to DCs. Bennett et al. reported that CD40L on helper T cells plays an crucial role in licensing DCs (43). Activated iNKT cells express CD40L transiently (44), but other lymphocytes do not. In fact, the adjuvant effect of DCs triggered by iNKT cells is eliminated when CD40^−/−^ mice are used as recipients (44), even though the iNKT cell response is still present. In a reciprocal study, co-administration of antigen plus soluble TNF-α and soluble IFN-γ induces the phenotypic maturation of DCs *in situ*, but does not generate antigen-specific T cells. Thus, phenotypic maturation of DCs does not always correspond to an antigen-specific T cell response, whereas functional maturation of DCs does. CD40–CD40L interaction during DC-iNKT cell cross talk is critical for DC maturation, resulting in IL-12 production (Figure 2), whereas inflammatory cytokines (TNF-α and IFN-γ) serve as co-factors for full maturation of DCs. In addition, iNKT cell-licensed DCs apparently use a different mechanism rather than that used during TLR signaling. The adjuvant effect of TLR ligands depends on either MyD88 or TRIF (45), or both, but that by iNKT-licensed DCs does not (32). As discussed above, NKT licensed-DCs depend on CD40/CD40L signaling and inflammatory signals (TNF-α and IFN-γ). CD40/CD40L signaling may involve the TRAFs (TRAF1, 2, 3, 5, 6), whereas TNF-α and IFN-γ signaling may involve the TRAF2 and JAK1/2-STAT1 pathway respectively (46, 47).

The location of DCs and iNKT cells in spleen is another important factor in their mutual activation. After their activation, iNKT cells accumulate in the marginal zone, where they co-interact with DCs. After activation by iNKT cells, XCR1^+^ DCs can traffic to the PALS area and then prime T cells (48). These responses are orchestrated by chemokines, cytokines, and cell surface molecules. iNKT cell-licensed DCs produce CCL17 (37, 49), which attracts CCR4^+^ CD8^+^ T cells for subsequent activation.

Several factors during the initiation of innate immunity determine the subsequent flavor of the adaptive immune response: (1) the number and function of iNKT cells and APCs, (2) the nature of the ligands (i.e., OCH, α-GalCer, or α-C-GalCer) (35), (3) the properties of host APCs (DC location or subset) (44), and (4) the level of CD1d expression (50). Thus, the magnitude of the innate immune response generated by all these factors can be directly correlated with the subsequent adaptive immune response.

## Development of iNKT-Triggered Antitumor Strategies Linking Innate and Adaptive Therapy

### Cellular Vaccines Acting As Immunological Adjuvant and Tumor Antigen Carrying Vector

We and others demonstrated that co-administration of antigen and iNKT ligand generates antigen-specific T cells, in addition to activating iNKT and NK cells (32, 33, 51). However, several optimal conditions are limited. It has been reported that DCs cannot phagocytize antigen after their maturation. Indeed, administration of tumor antigen 4 h after an administration of NKT cell ligand did not lead to a T cell response (32, 33). Therefore, tumor antigen and NKT cell ligands have to be delivered simultaneously to *in vivo* DCs (32, 33). In addition, we also found that this co-administration protocol generates CTL, but not memory T cells easily.

CD1d^+^ cells loaded with α-GalCer can activate iNKT cells directly *in vitro*, but it was not known whether adaptive immunity was generated after initiating this innate immune response. We first showed conclusively that CD1d^+^tumor/Gal can induce antigen-specific CD4^+^ and CD8^+^ T cell immunity (50) (Figure 1). From this finding, we proposed a strategy using all-in one cell type that expressed tumor antigen as well as CD1d that was loaded with the NKT cell ligand α-GalCer simultaneously, a cell that we reported an adjuvant vector cell (50, 52) (Figure 1). CD1d is generally expressed on most hematopoietic cells, e.g., DCs, B cells, T cells, and macrophages, and on some non-hematopoietic cells, e.g., intestinal epithelium and hepatocytes, including multiple tumor types (53). Therefore, approaches using adjuvant vector cells may be applicable not only for most hematological disorders, where they can be applied relatively easily, but also for many solid tumor cells, a more difficult therapeutic target. In fact, the therapeutic strategy using tumor/Gal has been extended to many types of CD1d^+^ tumor cells, including in our own studies, e.g., B16 melanoma, EL4 thymoma, WEHI3B leukemia, and J558 plasmacytoma. In addition, Hunn et al. reported that irradiated Glioma/Gal was effective in a prophylactic setting and also in a therapeutic setting together with Treg depletion of intracranial glioma model (54). Kobayashi et al. have reported on an α-GalCer-loaded B cell lymphoma (Eμ-myc tumor) combined with an agonistic antibody targeting 4-1BB (CD137) (55). The studies as above demonstrated the antigen-specific effector T cell-mediated survival. These tumor/Gal vaccines would be useful in an autologous setting.

Instead of such syngeneic tumor/Gal therapy, we newly established the concept of an artificial adjuvant vector cell (aAVC) as a new type of cancer vaccine platform that incorporates *in vivo* iNKT-licensed DC therapy (Figure 1). These cells (aAVC), NIH3T3 cells for mouse and HEK293 for humans, have been transfected with a CD1d and tumor antigen mRNA and then loaded with α-GalCer (37, 42, 56). The aAVC express the α-GalCer-CD1d complex on their surface and tumor antigen protein inside of the allogeneic cells. The aAVC treatment reduces the number of metastases, and eliminated grossly large tumors (37, 42, 56).

As the mechanism of adjuvant vector cells (tumor/Gal or aAVC), four immunological steps take place (Figure 2). Initially, these cells directly activate iNKT cells. iNKT cells producing IFN-γ can then simultaneously activate NK cells. These innate killer iNKT/NK cells capable of producing IFN- γ reject the adjuvant vector cells, but some of the killed adjuvant vector cells are taken up by DCs *in situ*, thereby several immunogenic features of DCs are engaged. The adjuvant vector cells-capturing DCs in lung, liver, and spleen become matured by their interaction with iNKT cells, resulting from CD40L–CD40 interactions and production of inflammatory cytokines. Next, the mature DCs present the TAAs to T cells on both MHC class I and II *in situ*. Particularly, the XCR1^+^DCs are specialized to cross-present antigens on MHC class I. Notably, when mice were vaccinated with adjuvant vector cells, they became resistant to the parental tumor cells. In fact, administration of adjuvant vector cells induces CTL and long-term memory T cells efficiently *in vivo* (48, 50, 52).

### Bacteria-Based Adjuvant Therapy

*Listeria monocytogenes* (LM) is a Gram-positive intracellular bacterium. Several groups have investigated whether recombinant LM lacking virulence genes, but expressing several TLR ligands such as lipoteichoic acid, would be useful for delivering TAA *in vivo* (57, 58). After infecting the target cells with LM, there was active phagocytosis and lysis of the bacteria in the phagosome. The recombinant LM allowed for the delivery of the TAA directly into macrophages and DCs, which can present TAA peptides to CD4^+^ and CD8^+^ T cells. In practice, a live attenuated, LM-based tumor vaccine expressing TAA-Mage-b (Mb) and α-GalCer has been studied (59) (Figure 1). The T cell-mediated antitumor efficacy resulting from direct incorporation of α-GalCer into live LM-Mb was found to be more powerful and safer than co-administration of the LM-Mb vaccine and α-GalCer, but the iNKT cell response was weaker.

Bacille Calmette–Guerin (BCG) was derived by attenuating *Mycobacterium bovis* and is widely used in many countries as a tuberculosis vaccine, although its efficacy has been contested. Recombinant BCG (rBCG) strains expressing either Listeriolysin-O from LM or perfringolysin from *Clostridium perfringens* have been investigated as candidate tumor vaccines (60). In simple rBCG-based vaccination models, skin CD11b^high^ DC subsets present antigen to CD4^+^ T cells (61). Using an approach of incorporating glycolipids into rBCG strains, rBCG strains expressing an SIV Gag antigen (rBCG-SIV gag) together with α-GalCer enhanced CTL more efficiently compared to responses primed by simple rBCG-SIV gag (62) (Figure 1). Similar to the concept of adjuvant vector cells or aAVC, these two types of bacteria vaccine expressing antigen and NKT ligand showed CTL induction more efficiently than that of co-administration approach.

## iNKT Cell Transfer Immunotherapy

As the other option, complementation of iNKT cell therapy may be an approach for cancer patients with decreased iNKT cell frequencies in target organs. This type of iNKT cell-based immunotherapy may be able to take advantage of the recent iPS reprograming technology. We recently established a protocol to reprogram human Vα24^+^iNKT cells and then to re-differentiate them into functional iNKT cells, so called iPS-iNKT cells (63). Similar to conventional human Vα24^+^iNKT cells, iPS-iNKT cells can produce IFN-γ upon NKT ligand activation and kill several types of human tumor cell lines (leukemia, lung cancer, and head and neck cancers) *in vitro* and *in vivo*. As already discussed in this review, we demonstrated that NK cell activity but not T cell induction is induced after the iNKT cell activation, mainly through IFN-γ production as iNKT–NK axis. In fact, once activated *in vivo*, human iPS-iNKT cells have been shown, in “humanized” NOG mice with human peripheral blood cells, to mediate adjunctive activity by activating autologous NK cells (Figure 1). We, therefore, suggest that human iPS-Vα24^+^iNKT cells could exert antitumor activity *in vivo*.

## Conclusion

When iNKT cells are activated by the ligand, they subsequently have the power to activate NK cells by producing IFN-γ as iNKT–NK axis. In fact, several immunotherapies using autologous DC/Gal in clinical trials of solid tumor and hematological malignancies have indicated the importance of IFN-γ-producing iNKT cells and NK cell activation (Figure 1). Although iNKT cells can mature DCs *in vivo*, IFN-γ alone is not sufficient to DC maturation and T cell induction. Therefore, to further develop iNKT cell-mediated therapy, many groups have focused on the interaction between DC and iNKT cells. We summarized details of the mechanism of the interaction between these two cell types and also introduced several iNKT cell-triggered DC approaches that are candidates for potential new therapies for cancer treatment. Adjuvant vector cells, including Tumor/Gal or aAVC as well as non-somatic cell adjuvants (bacteria) are all candidates for iNKT-triggered DC mediated immunotherapy.

## Author Contributions

SF and KS conceptualized, wrote and edited the manuscript.

## Conflict of Interest Statement

The authors declare that the research was conducted in the absence of any commercial or financial relationships that could be construed as a potential conflict of interest.
